# Association of Thyroid Function With Lipid Profile in Patients With Metabolic Syndrome: A Prospective Cross-Sectional Study in the Indian Population

**DOI:** 10.7759/cureus.44745

**Published:** 2023-09-05

**Authors:** Priti Abha, J R Keshari, Seema R Sinha, Kumar Nishant, Rekha Kumari, Prem Prakash

**Affiliations:** 1 Biochemistry, Indira Gandhi Institute of Medical Sciences, Patna, IND; 2 Biochemistery, Indira Gandhi Institute of Medical Sciences, Patna, IND; 3 Pediatrics, Jawaharlal Nehru Medical College and Hospital, Bhagalpur, IND; 4 General Surgery, Indira Gandhi Institute of Medical Sciences, Patna, IND

**Keywords:** dyslipidemia, insulin resistance, waist circumference, subclinical hypothyroidism, hypothyroidism, metabolic syndrome

## Abstract

Introduction

Metabolic syndrome is a group of aberrant metabolic indicators including hypertension, dyslipidemia, impaired fasting blood glucose, and obesity. It has been reported that thyroid hormones have a strong influence on the cardiovascular system, and hypothyroidism has been linked to metabolic syndrome components. The objective of the study was to find out the association of thyroid function with lipid profile in patients with metabolic syndrome.

Methods

A prospective cross-sectional study was conducted in an apparently healthy adult population visiting the outpatient Department of Indira Gandhi Institute of Medical Sciences (IGIMS), Patna, Bihar, India. Metabolic syndrome was diagnosed according to the International Diabetes Federation (IDF) criteria. Fasting blood glucose, triglyceride, and HDL levels were tested using the enzymatic photometric method. Thyroid-stimulating hormone (TSH), free T4, free T3, and insulin assays were performed using chemiluminescence immunoassay (CLIA).

Results

Out of 197 subjects recruited, 86 (51 males and 35 females) were diagnosed with metabolic syndrome according to the IDF criteria, and the rest 111 without metabolic syndrome were considered to be the controls. The mean age of subjects with and without metabolic syndrome was 45.8±8.5 and 46.4±9.6 years, respectively. The prevalence of thyroid dysfunction in the present study was 22%. In subjects with metabolic syndrome, most of the clinical and hormonal parameters (waist circumference, waist-height ratio, fasting blood sugar, fasting insulin, triglycerides, T3, and TSH) were significantly higher (p<0.001) as compared to those without metabolic syndrome. In case of lipid profile, the triglycerides in those with metabolic syndrome (262.8±112.3 mg/dL) were significantly higher (p<0.001) than those without metabolic syndrome (137.9±19.01 mg/dL), while the serum levels of HDL were significantly higher (p<0.001) in group without metabolic syndrome (50.5±3.9 mg/dL) as compared to those with metabolic syndrome (43.4±5.2 mg/dL). Also, the TSH levels were significantly higher (p<0.001) in subjects with metabolic syndrome (5.3±3.4 μl/mL) as compared to those without metabolic syndrome (2.6±1.4 μl/mL). Among all the components of metabolic syndrome, waist circumference and HDL showed a significant strong positive correlation (r=0.51) with TSH, and systolic blood pressure (r=0.39), diastolic blood pressure (r=0.39), and fasting blood sugar levels (r=0.44) showed significantly moderate positive correlation with TSH levels. T4 (OR=8.82; 95% CI: 1.56-49.8) and TSH (OR=1.61; 95% CI: 1.19-2.18) levels were observed to have significantly higher odds as risk factors for metabolic syndrome.

Conclusion

There is a significant association of thyroid function with lipid profile in metabolic syndrome. It was observed that along with metabolic alterations, cardiovascular symptoms of hypothyroidism and subclinical hypothyroidism are possible. Therefore, while evaluating people with metabolic syndrome, it may be appropriate to look into how well their thyroid glands are functioning.

## Introduction

The term "metabolic syndrome" refers to a group of disorders that include weight gain, hypertension, an abnormal lipid profile with high triglyceride (TG) levels and low levels of high-density lipoproteins (HDLs), and higher fasting blood sugar (FBS) levels [1]. In the years to come, diabetes and cardiovascular illnesses are more likely to occur in patients with metabolic syndrome. In individuals with metabolic syndrome, thyroid impairment is frequent [1,2]. In India, there is clear evidence of a high incidence of the metabolic syndrome, which is expanding globally. The incidence increased globally from 1.1% in 1980 to 3.85% in 2015 over the past three decades. The global rate of mortality from high body mass index (BMI) increased by 28.3% between 1990 and 2015 [2]. Insulin resistance and metabolic syndrome are quite common in India. According to studies, the age-adjusted prevalence of metabolic syndrome in urban Indian populations was determined to be around 25% overall (around 31% in women and 18.5% in males) [2].

A cluster of interconnected metabolic disorders known as metabolic syndrome include central obesity, high TGs, low HDL, hypertension, and hyperglycemia [3]. In comparison to those without the syndrome, those with metabolic syndrome have a higher risk of cardiovascular disease, myocardial infarction, stroke, and death from these conditions. The primary pathophysiological factor driving the clustering is thought to be insulin resistance [4]. When insulin activity intensifies, enhanced lipolysis generates more fatty acids, and this further reduces insulin's antilipolytic impact. Another potential pathophysiologic explanation for the metabolic syndrome is leptin resistance. In general, increased synthesis of apolipoprotein (APO) B containing TG-rich very low-density lipoprotein (LDL) is a consequence of free fatty acid flow to the liver. As APO C III levels rise, lipoprotein lipase is inhibited, which increases TGs and raises the risk of atherosclerotic cardiovascular disease. Insulin secretion and/or clearance changes in response to a deficiency in insulin action, increasing or decreasing, respectively, to maintain euglycemia. As a result of an insulin secretion deficiency, this compensatory mechanism ultimately fails, causing type II diabetes mellitus to develop from impaired fasting glucose [5].

The majority of organ functions are influenced by thyroid hormones, which have widespread impacts [6]. The thyroid is crucial for controlling metabolism. The metabolism of glucose and lipids, blood pressure control, and energy expenditure are all impacted in various ways by thyroid hormone [7]. This hormone may be linked to metabolic syndrome because it acts as a general pacemaker for the metabolic process. An elevated risk of atherosclerotic heart disease is linked to both metabolic syndrome and thyroid disorders. The connection between thyroid dysfunction and the metabolic syndrome is poorly understood. There have only been a few modest studies [8].

The mechanism of normal T3, T4, and increased thyroid-stimulating hormone (TSH) in obese individuals with metabolic syndrome is unknown, but it has been hypothesized that this condition is linked to insulin resistance because of a defect in post-receptor signal transduction in target tissue [9]. A similar mechanism of thyroid receptor resistance may also be at work in these individuals. On the one hand, obesity affects thyroid hormone levels, whereas subclinical hypothyroidism promotes a sluggish metabolism that contributes to obesity. Therefore, it is unknown if the increase or decrease in thyroid hormone levels is a result of obesity (metabolic syndrome) or a cause.

Even though there are studies on thyroid functioning in people with metabolic syndrome, there is a scarcity of studies on the issue from northern India. Therefore, the objective of the study was to assess the thyroid status of individuals with metabolic syndrome and to determine their association.

## Materials and methods

This is a prospective cross-sectional study conducted in the Department of Biochemistry and Department of General Medicine, Indira Gandhi Institute of Medical Sciences (IGIMS), Patna, Bihar, India. The study included 197 apparently healthy subjects over the age of 30 years who met the inclusion and exclusion criteria and visited the Department of General Medicine as outpatients and inpatients during a 24-month period. Metabolic syndrome cases were identified by applying the International Diabetes Federation (IDF) criteria. Detailed medication history, blood pressure, weight, height, BMI, and waist circumference were recorded in a case record form.

Inclusion criteria

Apparently healthy adults aged over 30 years visiting the Outpatient Department of General Medicine without any history of any systemic or chronic illness were included in the study. The subjects did not have any exposure to iodine or a contrast agent during the preceding three months. Subjects from both urban and rural areas were included in the study. Subjects who did not have any metabolic syndrome according to the IDF criteria were considered as controls.

Exclusion criteria

Subjects below 30 years, who were pregnant, and who had exposure to iodine or a contrast agent during the preceding three months were excluded from the study. Hemolyzed and lipemic samples were excluded from the study.

Ethical approval

The study was initiated after approval from the Institutional Ethics Committee, IGIMS (1938/IEC/IGIMS/2020). Proper informed consent was obtained before enrolling the subjects in the study.

Blood sample collection and processing

After a noted 8 to 10 hours of fasting, venous blood sample was drawn for measuring thyroid profile, lipid profile, insulin, and FBS. The blood sample collected was allowed to clot by placing in a rack at room temperature for at least 30 minutes and maximum for 1 hour. Then it was centrifuged at 3,000 rpm for 5 minutes, and the separated serum sample was stored at -20 degrees Celsius until used.

Biochemical analysis

The clear serum obtained from the whole blood was analyzed for TSH, free T3, free T4, and insulin on Abbott ARCHITECT i2000SR (Abbott, Chicago, IL) using the chemiluminescent microparticle immunoassay (CMIA) technology. Lipid profile and FBS were analyzed on Backman Coulter automated analyzers AU5800 and AU700 (Beckman Coulter, Inc., Crea, CA) by enzymatic photometry. The FBS and insulin were used for the calculation of homeostatic model assessment of insulin resistance (HOMA IR). All the assays were performed following the manufacturer's instructions strictly.

Statistical analysis

Statistical analysis was carried out using STATA Version 17 for Windows (Stata Corp., College Station, TX). Descriptive statistics were generated to enable comparisons between groups. Distributions were compared using Student’s t-test and ANOVA. Categorical variables were presented as numbers and percentages and were compared using the chi-square test. Pearson’s correlation was used to find out the relationship between different continuous variables. Multivariate logistic regression analysis was applied to evaluate the effect of thyroid function parameters on the presence of metabolic syndrome after adjusting for the confounders such as age, gender, waist circumference, and BMI. A p-value of <0.05 was considered statistically significant.

## Results

In a total of 197 subjects, 115 were males and 82 were females. Around 26.7% patients were in the age group of 30 to 39 years, 43.3% in the age group of 40 to 49 years, 21.1% in the age group of 50 to 59 years, and 8.9% were above 60 years of age.

Out of the 197 subjects, 86 (51 males and 35 females) were diagnosed with metabolic syndrome and 111 (64 males and 47 females) were healthy controls. The mean age of patients with and without metabolic syndrome was 45.8±8.5 and 46.4±9.6 years, respectively. In subjects with metabolic syndrome, most of the parameters (waist circumference, waist-height ratio, FBS, fasting insulin, TGs, T3, and TSH) were significantly higher (p<0.001) as compared to those without metabolic syndrome.

In case of lipid profile, TGs in those with metabolic syndrome (262.8±112.3 mg/dL) were significantly higher (p<0.001) than those without metabolic syndrome (137.9±19.01 mg/dL), while the serum levels of HDL were significantly higher (p<0.001) in patients without metabolic syndrome (50.5±3.9 mg/dL) as compared to patients with metabolic syndrome (43.4±5.2 mg/dL). Also, the TSH levels were significantly higher (p<0.001) in patients with metabolic syndrome (5.3±3.4 μl/mL) as compared to patients without metabolic syndrome (2.6±1.4 μl/mL) (Table 1).

**Table 1:** Characteristics of the study population with and without metabolic syndrome. BMI, body mass index; HOMA-IR, homeostatic model assessment of insulin resistance; HDL, high-density lipoprotein; TSH, thyroid-stimulating hormone

Characteristics	Metabolic syndrome (n=86)	Without metabolic syndrome (n=111)	P-value
Height (cm)	157.4±9.3	163.3±7.0	<0.001
Weight (kg)	75.3±12.2	72.5±11.6	0.104
BMI (kg/m^2^)	30.4±3.9	27.1±3.1	<0.001
Waist circumference (cm)	97.7±4.0	86.9±6.5	<0.001
Waist-height ratio	0.48±0.06	0.44±0.06	<0.001
Systolic blood pressure (mmHg)	140.9±12.2	122.9±6.3	<0.001
Diastolic blood pressure (mmHg)	88.3±5.2	77.5± 4.8	<0.001
Blood sugar (fasting) (mg/dl)	112.9±9.4	94.5±7.6	<0.001
Insulin (fasting) (mIU/mL)	20.8±4.3	5.4±3.9	<0.001
HOMA-IR	5.8±1.3	1.3±1.1	<0.001
Serum triglycerides (mg/dL)	262.8±112.3	137.9±19.0	<0.001
HDL (mg/dL)	43.4±5.2	50.5±3.9	<0.001
Free T4 (μg/dL)	1.09±0.42	1.08±0.19	0.868
T3 (μg/dL)	2.5±0.9	2.9±0.6	<0.001
TSH (μl/mL)	5.3±3.4	2.6±1.4	<0.001

The subjects were also stratified on the basis of thyroid dysfunction into three subgroups, namely, euthyroid, subclinical hypothyroid, and hypothyroid. A total of 154 subjects were in euthyroid group (free T4 and TSH level in range), 11 subjects in hypothyroid state (reduced free T4 and reduced TSH levels), and 32 in subclinical hypothyroid state (normal free T4 and raised TSH levels). Parameters such as weight, BMI, waist circumference, weight-height ratio, systolic blood pressure, and diastolic blood pressure were significantly lower (p<0.001) in the euthyroid group as compared to hypothyroid and subclinical hypothyroid groups.

A total of 107 subjects had insulin resistance (HOMA-IR, 5.9±1.1) whereas 90 subjects had insulin sensitivity (HOMA-IR, 1.06±0.39). Subjects with HOMA-IR>2.5 were considered to have insulin resistance. The hypothyroid (6.9±1.1) and subclinical hypothyroid groups (5.4±1.9) had significantly higher (p<0.001) insulin resistance as compared to the euthyroid group (2.6±2.3). The euthyroid subgroup (99.9±11.9 mg/dL) had significantly lower (p<0.001) mean FBS levels as compared to the hypothyroid (111.6±10.3 mg/dL) and subclinical hypothyroid groups (113.1±5.9 mg/dL).

In case of lipid profile, the TG levels in euthyroid subgroup (171.2±77.8 mg/dL) were significantly lower (p<0.001) as compared to the hypothyroid (356.3±141.1 mg/dL) and subclinical hypothyroid groups (238.2±100.2 mg/dL), while the serum levels of HDL were significantly higher (p<0.001) in the euthyroid group (48.6±5.1 mg/dl) as compared to the hypothyroid (42.6±6.9 mg/dL) and subclinical hypothyroid groups (43.1±5.5 mg/dL).

TSH levels were significantly higher (p<0.001) in the hypothyroid group (12.4±2.5 μl/mL) as compared to the euthyroid (2.6±1.11 μl/mL) and subclinical hypothyroid groups (6.4±1.8 μl/mL). T3 (p=0.04) and T4 levels (p<0.001) were significantly higher in the euthyroid group as compared to the subclinical and hypothyroid groups (Table 2).

**Table 2:** Characteristics of the subject population as per the thyroid status. BMI, body mass index; HOMA-IR, homeostatic model assessment of insulin resistance; HDL, high-density lipoprotein; TSH, thyroid-stimulating hormone

Characteristics	Euthyroid (n=154)	Subclinical Hypothyroidism (n=32)	Hypothyroid (n=11)	P-value
Height (cm)	161.2±8.3	158.6±9.7	159.7±9.8	0.28
Weight (kg)	72.6±11.6	75.3±10.9	86.1±13.8	<0.001
BMI (kg/m^2^)	27.8±3.5	29.9±3.5	33.7±4.2	<0.001
Waist circumference (cm)	89.9±7.2	96.2±5.7	103.3±3.2	<0.001
Waist-height ratio	0.45±0.06	0.47±0.05	0.54±0.07	<0.001
Systolic blood pressure (mmHg)	127.9±10.3	140.3±15.3	142.7±18.9	<0.001
Diastolic blood pressure (mmHg)	80.7±6.6	86.9±7.7	89.8±5.9	<0.001
Blood sugar (fasting) (mg/dL)	99.9±11.9	111.6±10.3	113.1±5.9	<0.001
Insulin (fasting) (mIU/mL)	9.7±7.6	19.4±6.4	24.9±3.7	<0.001
HOMA-IR	2.6±2.3	5.4±1.9	6.9±1.1	<0.001
Serum triglycerides (mg/dL)	171.2±77.8	238.2±100.2	356.3±141.1	<0.001
HDL (mg/dL)	48.6±5.1	43.1±5.5	42.6±6.9	<0.001
Free T4 (μg/dL)	1.14±0.25	1.0±0.39	0.59±0.38	<0.001
T3 (μg/dL)	2.8±0.7	2.6±1.01	2.2±1.2	0.04
TSH (μl/mL)	2.6±1.11	6.4±1.8	12.4±2.5	<0.001

Pearson’s correlation coefficient analysis was used to determine the correlation of free T3, T4, and TSH levels with waist circumference, systolic and diastolic blood pressure, FBS, serum TGs, and HDL. A weak negative correlation of free T3 (r = -0.23, p<0.05) and T4 (r = -0.15, p<0.05) levels was observed with waist circumference. In case of TSH, it had a significant positive correlation with all the parameters studied except serum TGs (r= -0.36, p<0.05) (Table 3).

**Table 3:** Correlation of components of metabolic syndrome with T3, T4, and TSH. Pearson’s correlation coefficient (r value) was calculated, and correlation was considered to be significant when p-values were less than 0.05 (p<0.05*). HDL, high-density lipoproteins; TSH, thyroid-stimulating hormone

Parameter	Free T3	Free T4	TSH
Waist circumference	-0.23*	-0.15*	0.51*
Systolic blood pressure	-0.22*	0.2	0.39*
Diastolic blood pressure	-0.29*	0.04	0.39*
Blood sugar (fasting)	-0.25*	-0.05	0.44*
Serum triglycerides	-0.34*	-0.0	-0.36*
HDL	0.32*	-0.12	0.51*

Table 4 depicts the results of multivariate regression analysis conducted to evaluate the potential of thyroid function parameters as independent risk factors for metabolic syndrome. T4 (OR=8.82; 95% CI: 1.56-49.8) and TSH (OR=1.61; 95% CI: 1.19-2.18) levels were observed to have significantly higher odds as risk factors for metabolic syndrome, while T3 levels were observed to be protective with an OR of 0.54 (Table 4).

**Table 4:** Multivariate regression analysis for the association between metabolic syndrome and thyroid function parameters. Multiple logistic regression analysis was conducted to evaluate the potential of thyroid function parameters as independent risk factors for metabolic syndrome. The regression model was adjusted for age, gender, waist circumference, and BMI OR, odds ratio; CI, confidence interval; BMI, body mass index

Independent variable	Adjusted OR (95% CI)	P-value
T3	0.54 (0.31-0.95)	0.033
T4	8.82 (1.56-49.8)	0.014
TSH	1.61 (1.19-2.18)	0.002

## Discussion

One of the most prevalent disorders among people with metabolic syndrome is thyroid dysfunction. The metabolic syndrome characteristics are impacted by thyroid hormones because they are crucial in controlling energy balance, glucose metabolism, and lipid metabolism [10,11]. The incidence of non-communicable diseases is quite high in patients with metabolic syndrome. Patients with metabolic syndrome are more likely to develop diabetes, cardiovascular disease, and thyroid disease in the future [10]. Several recent investigations have shown a link between metabolic syndrome and thyroid dysfunction. Thyroid dysfunction is characterized by changes in the levels of T3 and T4, as well as TSH. Metabolic syndrome has been shown to be two times more common in people with elevated TSH levels [10]. The risk of coronary heart disease was also observed to be enhanced in subclinical hypothyroidism with elevated TSH levels. In hypothyroidism, dyslipidemia and atherosclerosis are both prevalent [10]. HDL, LDL, and cholesterol levels are influenced by thyroid hormones, whereas TGs are impacted by TSH. Substantiating these observations, in the present study, we also observed a significant association of thyroid hormone levels with factors of metabolic syndrome.

The mean waist circumference of cases with metabolic syndrome was 97.7±4 cm in the current study. This observation was in contrast to the results of Saluja et al's study, which reported a mean waist circumference of 92.04±13.21 cm [12], Deshmukh et al.'s study, which reported a mean waist circumference of 98.6±9.7 cm [11], Chakradhar et al.'s study, which reported a mean waist circumference of 85.6±6.38 cm [10], and Ogbera et al.'s study, which reported a mean waist circumference of 93.5±14.1 cm [13]. When compared to the other metabolic syndrome indicators, Agarwal et al. showed that women with thyroid dysfunction had a greater waist circumference [14]. According to the findings of all these investigations, individuals with increased waist circumference have first undergone screening for metabolic syndrome and then thyroid dysfunction. In the present study, we also observed a significant positive correlation of waist circumference with TSH levels and a negative correlation with free T3 and T4 levels, implying an association of thyroid hormone status with waist circumference. Therefore, patients with increased waist circumference should be screened for both metabolic syndrome and thyroid dysfunction. In the present study, patients with metabolic syndrome had mean systolic blood pressure and diastolic blood pressure of 140.9±12.2 and 88.3± 5.2 mmHg, respectively. Meher et al reported similar outcomes [15]. A similar research was conducted by Aldhafiri et al., and the results showed that the mean systolic blood pressure and diastolic blood pressure were 130.44±2.82 and 88.17± 2.82 mmHg, respectively [16]. In the present study, we observed an elevated blood pressure in patients with metabolic syndrome as compared to patients without metabolic syndrome and a strong positive correlation of both systolic and diastolic blood pressure with TSH levels and a negative correlation with T3 levels. This observation implies a cardiovascular link with metabolic syndrome and thyroid dysfunction. Therefore, patients with blood pressure issues should also be closely monitored for metabolic syndrome and thyroid dysfunction.

The mean insulin and HOMA-IR index in our patients with metabolic syndrome were 20.8±4.3 mU/mL and 5.8±1.3, respectively. Almost similar levels of mean insulin and HOMA-IR index were reported by Aldhafiri et al. in patients with metabolic syndrome [16]. Compared to the findings of the current study, almost similar levels of FBS, serum TG, and HDL were reported by Khatiwada et al. [17].

In the current study, 22% of those with metabolic syndrome had thyroid impairment. The frequency of thyroid dysfunction in individuals with metabolic syndrome ranges from 21% to 51% according to several other studies from India, Nepal, the Middle East, Africa, and Europe [11,18-22].

Around 154 participants had normal thyroid function, 11 had hypothyroidism, and 32 had subclinical hypothyroidism, according to the thyroid function status. Most of the values related to metabolic syndrome were considerably lower in the euthyroid subgroup than those in the hypothyroid and subclinical hypothyroid groups. In our study, individuals with metabolic syndrome had TSH levels that were in the upper normal range, which may indicate some thyroid dysfunction in these patients. The majority of earlier research showed that metabolic syndrome and higher blood TSH levels were related. Therefore, patients with thyroid dysfunction, especially euthyroid, should be thoroughly examined for metabolic syndrome and managed accordingly. The mean TSH level of a healthy control population was 2.35±1.07 mIU/L in a case-control study examining risk factors for cardiovascular disease in an eastern Nepalese community, which is lower than the mean TSH of the current research [23].

The relationship between metabolic syndrome variables and the hypothyroid subgroup varies throughout studies and seems to depend on the patients' age, gender, and race. In the present study, only a weak but significantly inverse correlation of free T3 and T4 with waist circumference was found. With the exception of serum lipids, TSH had a strong positive correlation with other metabolic parameters studied. Waist circumference revealed a substantial positive link with TSH and a significant negative correlation with T3 and T4 among all the metabolic syndrome factors. Waist circumference was considerably different between individuals with and without thyroid disease in a study by Khatiwada et al., and HDL showed a significant inverse relationship with TSH level [17]. The components of metabolic syndrome may be influenced by thyroid hormones and thereby may affect lipid metabolism. TSH and TG have a positive relationship, whereas HDL and TSH have a negative relationship [17]. Roos et al. found free T4 to be substantially correlated with total cholesterol, LDL, HDL, and TG [24]. According to a research conducted by Park et al., TSH and T4 levels were linked to patients' waist size, blood pressure, glucose levels, and lipid levels. Changes in TSH levels were substantially correlated with increases in systolic and diastolic blood pressure, total cholesterol, and serum TG. Changes in diastolic blood pressure, serum TG, HDL, and fasting glucose were significantly associated with changes in free T4 level [25]. Subclinical hypothyroidism and metabolic syndrome were shown to be highly correlated in an Indian investigation, and there was evidence of a linear relationship between TSH levels and total cholesterol, TG, LDL, and HDL levels in the metabolic syndrome group [15]. TSH, however, was not associated with any of the metabolic syndrome markers in a study conducted in Turkey [26].

The results of a study conducted by Chakradhar et al. demonstrated a strong correlation between waist circumference and T4 and TSH. It demonstrates how common hypothyroidism and subclinical hypothyroidism are in metabolic syndrome patients, both of which may be harmful to cardiovascular health. Hypertension raises the risk of cardiovascular disease, while hypothyroidism increases lipid levels [10]. There was a strong inverse relationship between total cholesterol, TGs, and T3 levels in a study by Sharma et al. [27]. In addition, among 11,554 participants aged 45 to 79 years, those with subclinical hypothyroidism had significantly higher levels of total cholesterol, LDL cholesterol, and TGs, and lower levels of HDL cholesterol C, according to the European Prospective Investigation in Cancer and Nutrition-Norfolk prospective population study [28]. After correcting for gender, age, and the HOMA-IR, Lai et al. found in a Chinese cohort that there was a positive correlation between serum TSH levels within the normal range and serum TG levels [29].

In the current study, we observed that after adjusting for confounders such as age, gender, and BMI, T4 and TSH levels were found to be independent predictors of risk of metabolic syndrome. Although the present study advocates for an association of T4 and TSH levels with metabolic syndrome, there are contradictory studies that report no association of T4 and TSH levels with metabolic syndrome. In a study conducted in the Korean population, the authors reported no association of T4 and TSH levels with metabolic syndrome, which is in contrast to the findings of the current study [30]. In view of the findings of the present study, we strongly recommend that subjects with thyroid dysfunction may be screened for metabolic syndrome and vice-versa. Evaluating the thyroid function in patients with metabolic syndrome may help identify and prevent the risk of cardiovascular and cerebrovascular events in the patients.

Limitations of the study

The small sample size and the single-center nature of the study were the major limitations of the study. Majority of the population was rural with mixed food habits. However, none of them used ultra-processed food or any other food supplement. Therefore, larger multi-center studies including participants with different ethnic backgrounds and eating patterns should be conducted to further generalize the findings of the study.

## Conclusions

Our study concluded that thyroid status and metabolic syndrome are associated with each other, and this association may raise the risk of cardiovascular and cerebrovascular events. Thyroid dysfunction was common among individuals with metabolic syndrome. Studies like ours will have a prognostic significance for clinicians in their routine clinical practice to develop strategies for better treatment and management. Further multi-center studies with large sample sizes are warranted.
